# Gallium single-photon emission computed tomography/computed tomography–guided treatment of outflow graft infection during left ventricular assist device support

**DOI:** 10.1016/j.xjtc.2021.08.013

**Published:** 2021-08-19

**Authors:** Shohei Yamada, Satoshi Kainuma, Koichi Toda, Shinichiro Tahara, Yoshiki Sawa

**Affiliations:** a Department of Cardiovascular Surgery, Osaka University Graduate School of Medicine, Osaka, Japan; b Department of Pathology, Osaka University Graduate School of Medicine, Osaka, Japan

**Figure:**
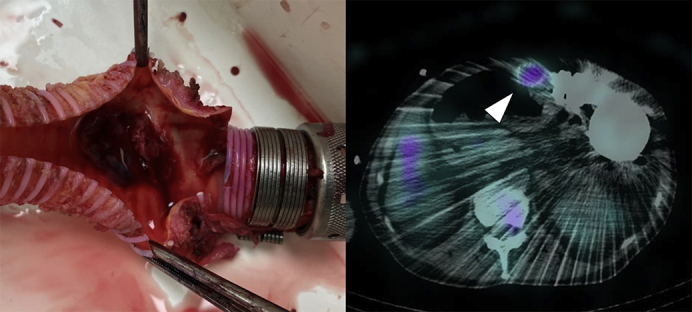
Outflow “endograft” infection detected in a patient with left ventricular assist device.

**Central Message**

Gallium single-photon emission computed tomography/computed tomography is a useful clinical modality for the treatment of outflow “endograft” infection in patients with left ventricular assist device.

See Commentary on page 356.

Device-related infection is a major complication of implantable left ventricular assist device (LVAD).^1^ Gallium single-photon emission computed tomography/computed tomography (Ga-SPECT/CT) can be useful in diagnosing device-related infections in patients with LVADs.^2^^,^^3^ We herein report a rare case of the LVAD outflow “endograft” infection in which Ga-SPECT/CT was used to identify the source of infection and determine the surgical strategy. The institutional review board of Osaka University Hospital approved the study protocol and publication of data (number 16105; date: February 11, 2016). The patient provided informed written consent for the publication of the study data.

## Clinical Summary

A 54-year-old man with dilated cardiomyopathy who had been supported with EVAHEART (Sun Medical Technology Research Corp, Nagano, Japan) for bridge-to-transplantation for 2.5 years underwent pump exchange surgery via lateral thoracotomy due to pump dysfunction. One month later, he developed a driveline infection, spreading into the pump pocket. Translocation of the pump (into intra-abdominal space) and omentopexy were performed. The patient remained well as an outpatient for 8 months until readmission for high fever. Blood culture was positive for *Pseudomonas aeruginosa*. Intravenous antimicrobial therapy was immediately started, which was effective in controlling the infection and improving the patient's physical condition. Two months later, the patient presented with fever associated with refractory bacteremia and subsequently developed massive cerebral hemorrhage, leading to left hemiplegia. Emergency craniotomy was performed, and his condition stabilized. However, 2 months later, the patient developed recurrent intracranial hemorrhage associated with bacteremia.

**Figure 1:**
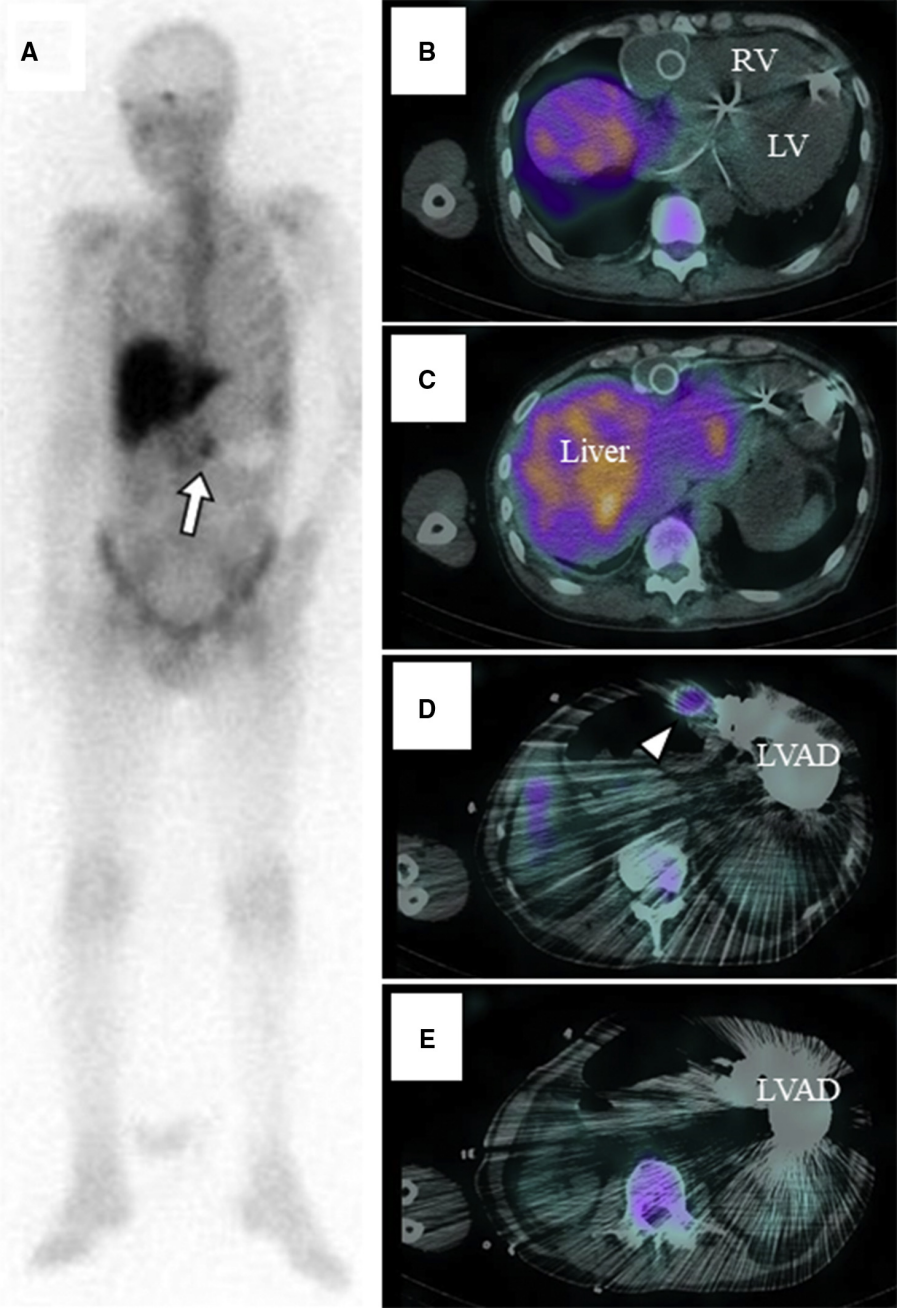
A, Gallium scintigraphy showed focal accumulation on the right side of the LVAD pump (*white arrow*). B-E, The site in which Ga-SPECT/CT accumulated was coincident with the outflow graft (*white arrowhead*). In addition, we could not find any uptake at the LVAD pump, pump pocket, driveline, and outside of outflow graft. *RV*, Right ventricle; *LV*, left ventricle; *LVAD*, left ventricular assist device.

**Figure 2:**
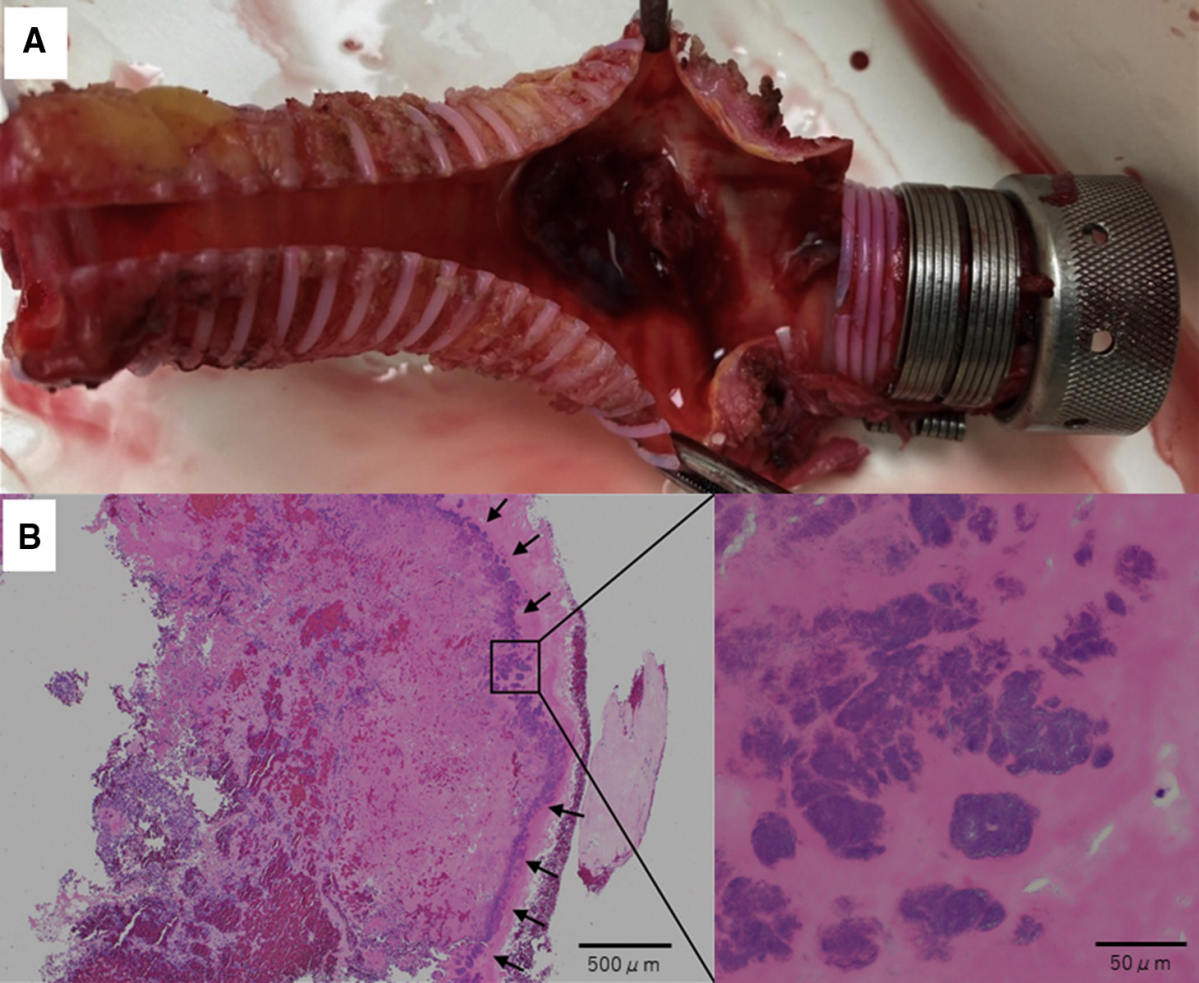
A, The vegetation with thrombus was found inside the outflow graft at the location, where Ga-SPECT/CT imaging accumulated. The culture of the vegetation was positive for *Pseudomonas aeruginosa*, which is consistent with the pathogen of the bacteremia. B, Pathologic examination revealed bacterial colonies and inflammatory cells in the vegetation on the outflow graft (*black arrows*).

Endocarditis was ruled out on transesophageal echocardiography. Then, Ga-SPECT/CT revealed a localized accumulation inside the proximal outflow graft, whereas no uptake was found in other LVAD components (Figure 1). To control the infection, partial replacement of the outflow graft was planned. The proximal outflow graft adhered to surrounding tissues in which bacteria were not detected in smear preparation. As there was concern on the worsening of intracranial hemorrhage related to completely heparinized cardiopulmonary bypass, a closed-circuit cardiopulmonary bypass via femoral cannulations was established, for which the amount of heparin was reduced to strictly control activated clotting time (ie, approximately 180 seconds). The outflow graft was distally crossclamped and transected, while the proximal part of the graft was longitudinally opened, revealing vegetation with thrombus inside the graft (Figure 2, *A*). The vegetation was sent for culture and was positive for *P aeruginosa*. The infected part of the graft was resected, and a new outflow graft was engaged with the pump and anastomosed to the previous one (Video 1). Pathologic examination revealed the presence of bacterial colonies within the organized thrombus (Figure 2, *B*). A year later, a heart transplant was performed successfully.

**Video 1:**
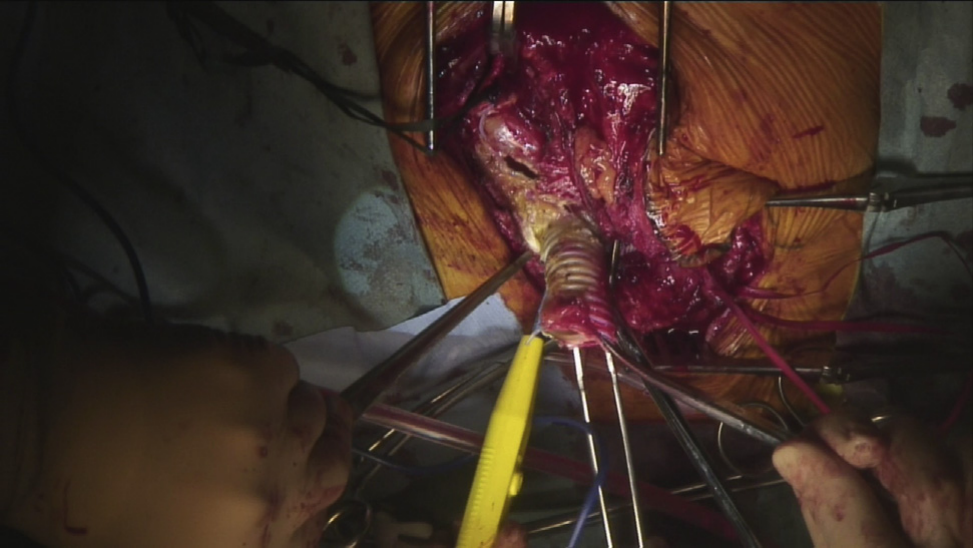
Partial replacement of the outflow graft. After we confirmed a negative result in smear preparation and established closed-circuit cardiopulmonary bypass via femoral cannulations, the outflow graft was distally crossclamped and transected. The proximal side was disconnected at the joint to the pump. The infected part of the graft was resected, then a new outflow graft was engaged with the pump and anastomosed to the previous one. Video available at: https://www.jtcvs.org/article/S2666-2507(21)00568-X/fulltext.

## Discussion

Nuclear medicine imaging is a promising modality to confirm the presence and extent of device-related infections. Levy and coworkers^3^ reviewed 5 patients suspected of device-related infections and demonstrated the utility of Ga-SPECT/CT in diagnosing LVAD pump (pocket) infection mainly derived from deep driveline infection. Our case is unique and different from those cases from the viewpoint of developing internal infection of outflow graft without any driveline infection or pump-pocket infection. It is worth noting that the presence of bacteria, which was pathologically confirmed inside the outflow graft, corresponds to the site at which the uptake was shown on Ga-SPECT/CT. This type of infection was difficult to diagnose without Ga-SPECT/CT due to its location inside the graft, having no external spread and abscess formation. In addition, we had to rule out pump-pocket infection due to the patient's medical history; Ga-SPECT/CT helped us by showing no uptake in any other LVAD components. As a result, the patient was able to undergo less-invasive surgery for complete removal of the infection source. Some might claim that it is difficult to completely exclude the possibility of the LVAD interior infection even in the absence of increased radiotracer activity around the pump. We would agree with the concern and consider if the patient had been in good health, replacement of the entire LVAD system would have been the alternative. As few data exist regarding sensitivity and specificity for imaging infection in implanted cardiac devices; we hope our case will stimulate further studies on this topic.

Interestingly, the location of the vegetation in the outflow graft was consistent with where it was clamped in the previous surgery. This indicates that clamping the outflow graft in the chronic phase might have damaged the intima, promoted thrombus formation, and caused risk for infection. In conclusion, Ga-SPECT/CT is useful in identifying the source of infection and could be one of the clinical modalities for guiding the treatment of outflow “endograft” infection in patients with LVADs.

## Supplementary Data

Video 1 Partial replacement of the outflow graft. After we confirmed a negative result in smear preparation and established closed-circuit cardiopulmonary bypass via femoral cannulations, the outflow graft was distally crossclamped and transected. The proximal side was disconnected at the joint to the pump. The infected part of the graft was resected, then a new outflow graft was engaged with the pump and anastomosed to the previous one. Video available at: https://www.jtcvs.org/article/S2666-2507(21)00568-X/fulltext.
